# Molecular Evidence of Orthomyxovirus Presence in Colombian Neotropical Bats

**DOI:** 10.3389/fmicb.2022.845546

**Published:** 2022-04-26

**Authors:** Manuel Uribe, Miguel E. Rodríguez-Posada, Gloria C. Ramirez-Nieto

**Affiliations:** ^1^Microbiología y Epidemiologia Research Group, Facultad de Medicina Veterinaria y de Zootecnia, Universidad Nacional de Colombia, Bogotá, Colombia; ^2^CIBAV Research Group, Veterinary Medicine School, Universidad de Antioquia, Medellín, Colombia; ^3^Research Center Fundación Reserva Natural La Palmita, Grupo de Investigaciones Territoriales Parael uso y Conservación de la Biodiversidad, Trinidad, Colombia

**Keywords:** Orthomyxovirus, Alphainfluenzavirus, bat-virus, influenza, Colombia

## Abstract

The Orthomyxoviridae family includes the genera Influenzavirus, Isavirus, Quaranjavirus, and Thogotovirus. In turn, Influenzavirus can be classified into four types: α, β, γ, and δ (Formerly A, B, C, and D), from which Alphainfluenzavirus (AIV) has the broadest host range, including birds, mammals, reptiles, and amphibians. Additionally, AIV has shown global epidemiological relevance owing to its pandemic potential. The epidemiological relevance of Chiropteran due to its multiple functional characteristics makes them ideal reservoirs for many viral agents. Recently, new influenza-like subtypes have been reported in Neotropical bats, but little is known about the relevance of bats as natural reservoirs of influenza viruses. Therefore, the current study aimed to determine the presence of AIV and new influenza-like subtypes in South American bats. For a better understanding of the drivers and interactions between AIV and bats, we used molecular assays with different gene targets (i.e., M, NP, and PB1) to identify AIV in New World bats. A housekeeping gene (CytB) PCR was used to check for nucleic acid preservation and to demonstrate the bat-origin of the samples. A total of 87 free-living bats belonging to 25 different species of the families Phyllostomidae and Vespertilionidae were collected in Casanare, Colombia. As a result, this study found seven AIV-positive bat species, three of them reported for the first time as AIV prone hosts. Neither of the AIV-like analyzed samples were positive for H_17_N_10_/H_18_/N_11_ subtypes. Although additional information is needed, the presence of a completely new or divergent AIV subtype in neotropical bats cannot be discarded. Collectively, the results presented here expand the epidemiological knowledge and distribution of AIV in neotropical free-ranging bats and emphasize the need to continue studying these viruses to establish the role they could play as a threat to animal and public health.

## Introduction

Orthomyxoviridae is a family of enveloped negative-sense single-stranded RNA (-ssRNA) viruses (Shaw and Palese, 2013) composed of seven genera. The Quaranjavirus and Thogotovirus, both Arboviruses transmitted by ticks, mainly infect wildlife (L’vov et al., 2014; Kosoy et al., 2015; Ballard et al., 2017), Isavirus causes infectious anemia in salmonids (Batts et al., 2017), and Influenzavirus, the epidemiologically more relevant genus, which includes four types: α, β, γ, and δ (formerly known as A, B, C, and D). These types are determined by specific characteristics of matrix protein 1 (M1) and the nucleoprotein (NP) genes (Palese and Young, 1982; Tobita, 1997; Sanjuan et al., 2009). Within these, Alphainfluenzavirus (AIV) have shown global epidemiological relevance (Messenger et al., 2014) owing to the pandemic potential (Taubenberger and Morens, 2010) and a wide range of susceptible hosts among wild birds and poultry (Causey and Edwards, 2008; Gonzalez-Reiche and Perez, 2012) terrestrial felines, canines, equids (Keawcharoen and Oraveerakul, 2004; Parrish et al., 2015; Zhou et al., 2015; Xie et al., 2016), swine and humans (Rose et al., 2013), as well as marine mammals (Ohishi et al., 2006; Blanc et al., 2009; Ramis et al., 2012; Boyce et al., 2013; Groth et al., 2014), the Amphibia and Reptilia Class (Mancini et al., 2004; Davis and Spackman, 2008; Temple et al., 2015), and bats in which new influenza-*like* subtypes have been reported (Tong et al., 2012, 2013). On the other hand, Beta and Gammainfluenzavirus cause a mild to moderate disease in humans (Taubenberger and Morens, 2008) and they can also infect seals and swine, respectively (Kimura et al., 1997; Osterhaus et al., 2000). Finally, Deltainfluenzavirus, the newest genus, was recently identified in ruminants and swine (Hause et al., 2014; Ferguson et al., 2015; Ng et al., 2015; Quast et al., 2015; Salem et al., 2017).

The order Chiroptera has approximately 1,224 species distributed all over the world (Wilson and Reeder, 2005; Fenton and Simmons, 2015), comprising approximately 25% of mammalian species and, thus, the second most biodiverse order in animal kingdom (Mickleburgh et al., 2002; Burgin et al., 2018). Bats are unique among mammals with remarkable diversity, global distribution, and accumulated flight distances of up to 2,518 km (Fleming and Peggy, 2006; Richter and Cumming, 2008). These characteristics together with unique anatomo-physiological, biological, and etiological features, make them ideal natural reservoirs and key pieces of the eco-epidemiological dynamics of several emergents and reemerging viral infectious diseases linked to human spillovers (Omatsu et al., 2007; Wong and Lau, 2009; Wang et al., 2011; Chan et al., 2013; O’Shea et al., 2014; Brook and Dobson, 2015; Han et al., 2015; Allocati et al., 2016; Plowright et al., 2016). Approximately 61% of human diseases are considered zoonotic and wildlife reservoirs are the source of most human emerging infectious diseases (Taylor et al., 2001; Childs et al., 2007). It is well-known that many viral pathogens have arisen through adaptation and/or cross-species transmission events. Bat-associated viruses database (DBatVir)^1^ and other authors report around 30 viral families identified in bat species (Table 1) (Chen et al., 2014). Otherwise, global search on bat viruses resulted in the detection and sometimes isolation of over 200 viruses from almost all viral families, thus, suggesting that bats may harbor substantial diversity of viruses rivaling or even surpassing viral diversity found in rodents (Misra, 2020). Despite decades of research into bats and associated pathogens, the bat-virus ecology and molecular biology remain still quite unexplored, with many questions largely unsolved (Letko et al., 2020). There have been several major bat-borne viruses outbreaks such as Hendra, Sosuga, Nipah, Marburg, and Ebola virus diseases, the severe acute respiratory syndrome (SARS-CoV), Middle East respiratory coronavirus (MERS-CoV), and the most recent SARS-CoV-2 virus responsible for the last pandemic, along with the report of two influenza-*like* viruses (Orthomyxoviridae) in South American bats, the H_17_N_10_/H_18_/N_11_ subtypes, which were identified by NGS methodologies in 2012. These two new bats-derived influenza-*like* viruses show different specific structural features affecting sialic acid receptor binding capability making them different from avian or human influenza viruses. To determine whether there is a risk for reassortment, and therefore a major concern about potential influenza pandemics originating from unknown bat origin viruses including orthomyxoviruses, it is necessary to establish the spectrum of viral diversity that exists in the mammalian species of order Chiroptera (Tong et al., 2013; Letko et al., 2020; Irving et al., 2021).

**TABLE 1:** Worldwide viral families reported in bat species.

Viral genome^¥^					
-ssRNA	+ssRNA	dsDNA	ssDNA	RT-Virus	dsRNA
Arenaviridae^§^	Astroviridae	Adenoviridae	Anelloviridae	Hepadnaviridae	Reoviridae
Bornaviridae	Caliciviridae	Asfarviridae^†^	Circoviridae	Retroviridae	Picobirnaviridae^‡^
Filoviridae	Coronaviridae	Herpesviridae	Parvoviridae		
Hantaviridae	Flaviviridae	Papillomaviridae			
Nairoviridae	Hepeviridae	Polyomaviridae			
Orthomyxoviridae	Picornaviridae	Poxviridae			
Paramyxoviridae	Togaviridae				
Peribunyaviridae					
Phenuiviridae					
Rhabdoviridae					

*^¥^Viral classification according to Koonin et al. (2021), ^§^Cogswell-Hawkinson et al. (2012), ^†^Hu et al. (2017), and ^‡^Dacheux et al. (2014). -ssRNA, negative-sense single-stranded RNA; +ssRNA, positive sense-single-stranded RNA; dsDNA, double stranded DNA; ssDNA, single stranded DNA; RT-Virus, reverse transcriptase; dsRNA, double-strand RNA.*

Despite advances to understand the viral dynamics of new influenza-*like* subtypes in bats (Dlugolenski et al., 2013; Aguiar et al., 2016; Ciminski et al., 2017) and characterization of different genomic segments (Garcia-Sastre, 2012; Sun et al., 2013; Zhu et al., 2013; Juozapaitis et al., 2014; Poole et al., 2014; Tefsen et al., 2014; Turkington et al., 2015; Hoffmann et al., 2016; Maruyama et al., 2016), the eco-epidemiology of those viral subtypes remains poorly understood due to sparse reports of influenza-like positive bat species and a lack of studies across Neotropics. Therefore, the present study aims to determine the presence of AIV and influenza-*like* subtypes in Colombian bats through molecular techniques as an approach to contribute to the knowledge of the neotropical distribution of this viral agent.

## Materials and Methods

### Studied Areas and Animal Sampling

**FIGURE 1:**
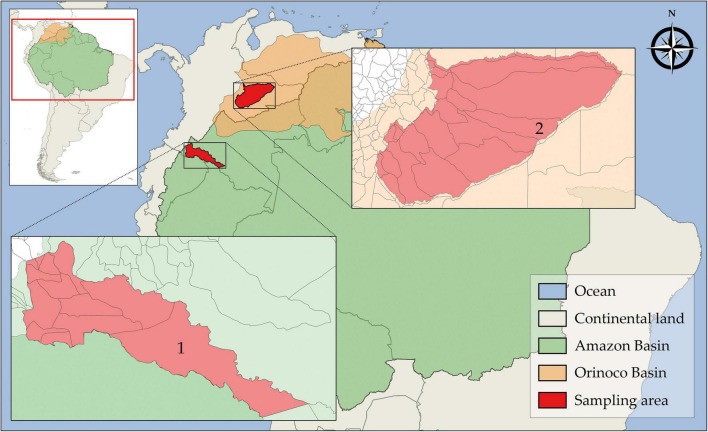
Geographic location of sampling areas in the Amazon and Orinoco Basins. (1) Puerto Leguízamo, Putumayo and (2) Trinidad, Casanare.

Different populations of free-ranging bats were investigated in two highly biogeographically divergent regions. The first sampling area was in the south-southwest amazon rainforest biome in the Putumayo department and the second was in the east-southeast floodable savannas of Orinoco Basin (Figure 1). The study was conducted from January 2016 to December 2017. A total of 87 wild free-ranging Yangochiroptera bats were captured by mist-netting and manually collected. Thereafter, the bats were morphologically identified and classified into species based on taxonomic keys on external and craniodental morphology by a specialist mastozoologist (Table 2). The collected bats were healthy and showed no signs of disease. Next, oropharyngeal, and rectal samples were collected separately by deep swabbing using CLASSIQSwabs™ (Copan) from each bat. Swab samples were preserved in FTA™ classic cards (Whatman™), and RNAlater™ (Invitrogen™), incubated at 4°C overnight, and stored at −80°C until further molecular assays were performed. Additionally, tissue samples were collected.

**TABLE 2:** Complete list of Yangochiroptera bat specimens collected.

Family	Genus	Species	Diet (food source)	(*n*)
Phyllostomidae	*Anoura*	*caudifer*	O	1
Phyllostomidae	*Artibeus*	*lituratus*	F	6
Phyllostomidae	*Artibeus*	*planirostris*	F	2
Phyllostomidae	*Carollia*	*brevicauda*	F/I	6
Phyllostomidae	*Carollia*	*castanea*	F/I	1
Phyllostomidae	*Carollia*	*perspicillata*	0	23
Phyllostomidae	*Desmodus*	*rotundus*	H	1
Vespertilionidae	*Eptesicus*	*chiriquinus*	I	2
Phyllostomidae	*Gardnerycteris* ^§^	*crenulatum*	C/I	4
Phyllostomidae	*Lonchophylla*	sp.	N	2
Phyllostomidae	*Lophostoma*	*brasiliense*	O	2
Phyllostomidae	*Mesophylla*	*macconnelli*	F	1
Vespertilionidae	*Myotis*	sp. nov.	I	3
Phyllostomidae	*Phyllostomus*	*discolor*	O	1
Phyllostomidae	*Phyllostomus*	*hastatus*	O	1
Phyllostomidae	*Phyllostomus*	*elongatus*	O	3
Phyllostomidae	*Platyrrhinus*	*brachycephalus*	F	7
Phyllostomidae	*Platyrrhinus*	*helleri*	F	1
Phyllostomidae	*Rhinophylla*	*fischerae*	F	4
Phyllostomidae	*Rhinophylla*	*pumilio*	F/I	2
Phyllostomidae	*Sturnira*	*tildae*	F	1
Phyllostomidae	*Sturnira*	*lilium*	F	7
Phyllostomidae	*Tonatia*	*saurophila*	O	2
Phyllostomidae	*Trachops*	*cirrhosus*	C/I	3
Phyllostomidae	*Vampyriscus*	*bidens*	F	1

*^§^Synonym Mimon sp. O, omnivorous; F, frugivorous; I, insectivorous; H, hematophagous; C, carnivorous; N, nectarivore.*

### Total Nucleic Acid Extraction

Total ribonucleic acid (TRA) from swabs was obtained using the high throughput QIAamp™ Viral RNA Mini Kit (Qiagen™) following the manufacturer’s instructions. Previous to the TRA extraction protocol from FTA™ card samples, 6 mm diameter disks were excised and incubated in TE buffer overnight (Sakai et al., 2015) at 4°C (Ndunguru et al., 2005) in absolute darkness. Likewise, RNAlater™ preserved swabs were temperate and gently homogenized by vortex in phosphate-buffered solution previous RNA extraction. All TRA samples were used immediately for molecular assays. High Pure Viral Nucleic Acid Kit (Roche™) was used for tissue total nucleic acid extraction.

### Housekeeping Gene Assay

Small mammal Cytochrome B gene was used as a housekeeping gene. For this purpose, a PCR method with a set of primers designed to amplify a 946 bp highly conserved region of this gene was used. The sequence of universal degenerated primers was: CytBUnifw 5′-TCATCMTGATGAAAYTTYGG-3′ and CytBUnirev 5′-ACTGGYTGDCCBCCRATTCA-3′, amplification conditions were adapted from Schlegel et al. (2012) and performed in a Labcycler Gradient (SensoQuest™ GmbH).

### Polymerase Chain Reaction Assays for Alphainfluenzavirus Detection

To assess AIV detection in oropharyngeal and rectal swabs, a fluorogenic real-time reverse transcription-polymerase chain reaction (RT-qPCR) targeting a highly conserved region of the M gene was applied. The set of primers and probes used were: IndiForward 5′-GACCRATCCTGTCACCTCTGAC-3′, InfAReverse 5′- AGGGCATTYTGGACAAAKCGTCTA-3′, and InfAProbe 5′-TGCAGTCCTCGCTCACTGGGCACG-3′ (CDC., 2009). The amplification procedure consisted of 30 min at 50°C, followed by 2 min at 95°C, 50 cycles for 15 s at 95°C, and 30 s at 55°C, where fluorescence was collected. Samples were tested by triplicate in a 25 μL final reaction volume. Negative, positive, and non-template controls were included in all tested plates. For quantification of viral load and measure of the Cq threshold, at least two standard template dilutions of known viral concentration from a cloned M segment were included. Unknown viral copy number in samples was calculated from the external curve of known concentration templates analyzed on the same plate.

Additionally, we carry out the detection of influenza-like subtypes by RT-qPCR and endpoint RT-PCR. The RT-qPCR target a 90 bp segment of the nucleoprotein (NP) gene. The reactions were performed using the following specific primers and probes: GTMFluNPFor, GTMFluNPRev, and GTMFluNP probe (Tong, 2015). Thermic amplification conditions were: 30 min at 45°C, followed by 5 min at 94°C, 40 cycles for 15 s at 94°C, and 60 s at 60°C. RT-qPCR assays were performed in a LightCycler™ 480 Instrument II (Roche™) using Super-Script III™ Platinum One-step (Invitrogen™). The TaqMan™ probes were labeled at 5′-end with the 6-carboxyfluorescein reporter and Blackhole Quencher 1 (Biosearch Technologies™) at 3′-end. Based on viral concentration expected in the low cell concentration of cell-free swab samples, an increase of fluorescent signal below the 37 Cq threshold was selected as the cut-off value (Bustin et al., 2009; Bustin and Nolan, 2017). All RT-qPCR AIV results are in agreement with MIQE guidelines (Bustin et al., 2009; Bustin and Nolan, 2017) and RDML data standard^2^ (Lefever et al., 2009).

The influenza-like PB1 gene-specific endpoint PCR, designed by Dr. Lucas Matías Ferreri (Department of Population Health, PDRC, UGA, United States) was performed after retrotranscription using Random Hexamer primers (Thermo Scientific™). The following set of PB1-specific primers was used: BatPB1-970For and BatPB1-1260Rev (available upon request). Reactions were performed in a Labcycler Gradient (SensoQuest™ GmbH) using the following amplification conditions: 2 min at 94°C, followed by 40 cycles for 15 s at 94°C, 15 s at 52°C, and 30 s at 72°C, with a final extension step of 30 s at 72°C. High sensibility SuperScript™ III One-Step RT-PCR System with Platinum™ Taq DNA Polymerase (Invitrogen™) was used. The pHW_Bat_NP and the pHW_Bat_PB1 DNA plasmid used as positive control were kindly provided by Dr.Daniel R. Pérez at the Department of Population Health, PDRC, UGA, United States.

## Results

### CytB-Polymerase Chain Reaction as Bat Housekeeping Gene and Preservation Control

**FIGURE 2:**
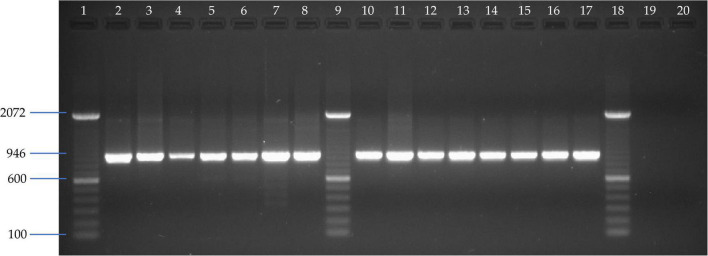
Agarose gel electrophoresis of CytB-specific PCR amplified fragments assay. Line 1, 9, and 18: 100 bp DNA ladder (Invitrogen); Line 2: *Vampyriscus bidens*; Line 3: *Anoura caudifer*; Line 4: *Desmodus rotundus*; Line 5: *Carollia brevicauda*; Line 6: *Carollia castanea*; Line 7: *Lonchophylla* sp; Line 8: *Gardnerycteris crenulatum*; Line 10: *Tonatia saurophila*; Line 11: *Sturnira tildae*; Line 12: *Myotis* sp; Line 13: *Trachops cirrhosus*; Line 14: *Platyrrhinus brachycephalus*; Line 15: *Artibeus lituratus*; Line 16: *Eptesicus chiriquinus*; Line 17: *Mesophylla macconnelli*; Line 19 and 20: Negative controls from MDCK cell line and allantoic fluid from chicken embryonated SPF eggs, respectively.

A total of 15 liver samples from different bat species of Phyllostomidae and Vespertilionidae bat families analyzed by CytB-PCR showed the expected 946 bp amplicon (Figure 2). Therefore, the results of this PCR assay from different bat species confirmed the chiropteran origin of the samples and the suitability of preserved nucleic acids for subsequent molecular assays.

### Alphainfluenzavirus RT-qPCR Assays

A total of 127 swab samples were analyzed by RT-qPCR in triplicates and given as positive when two or more replicates had a Cq less than or equal to 37 cycles. Quantification of cycle threshold (Cq) ranged from 34.17 to 37. Furthermore, the virus copy number per sample was determined using an eight-point standard curve. As a result, a total of 10 neotropical bat swab samples belonging to 7 different species were found positive for AIV by RT-qPCR assay (Table 3). The amount of AIV M gene segment and thus, the number of AIV viral particles by reaction varied from 4,51 × 10^2^ in insectivorous *Gardnerycteris crenulated* from Putumayo to 6,62 × 10^3^ in frugivorous *Carollia perspicillata* from Casanare.

**TABLE 3:** Alphainfluenzavirus (AIV) positive samples by RT-qPCR assay.

Species	Copy/reaction	OS	RS	R	F
*Gardnerycteris crenulatum*	4,51 × 10^2^	x	x	x	
*Carollia brevicauda*	1,88 × 10^3^		x		x
*Trachops cirrhosus*	1,87 × 10^3^		x		x
*Platyrrhinus brachycephalus*	1,95 × 10^3^		x		x
*Artibeus lituratus*	1,86 × 10^3^	x			x
*Artibeus planirostris*	6,32 × 10^3^	x			x
*Carollia perspicillata*	4,10 × 10^3^		x		x
*Artibeus lituratus*	6,30 × 10^3^	x			x
*Carollia perspicillata*	6,62 × 10^3^	x			x
*Carollia perspicillata*	5,09 × 10^3^	x			x

*OS, Oropharyngeal swab; RS, Rectal swab; R, RNAlater-preserved; F, FTA-preserved.*

### Alphainfluenzavirus-Like Tested Samples

Neither RT-qPCR targeting nucleoprotein (NP) nor endpoint PCR for the basic polymerase 1 (PB1) gene segments were detected. Therefore, none out of 127 samples tested showed amplification for these genes corresponding to NP and PB1 of the H_17_N_10_ and H_18_N_11_ influenza-like virus subtypes.

## Discussion

**FIGURE 3:**
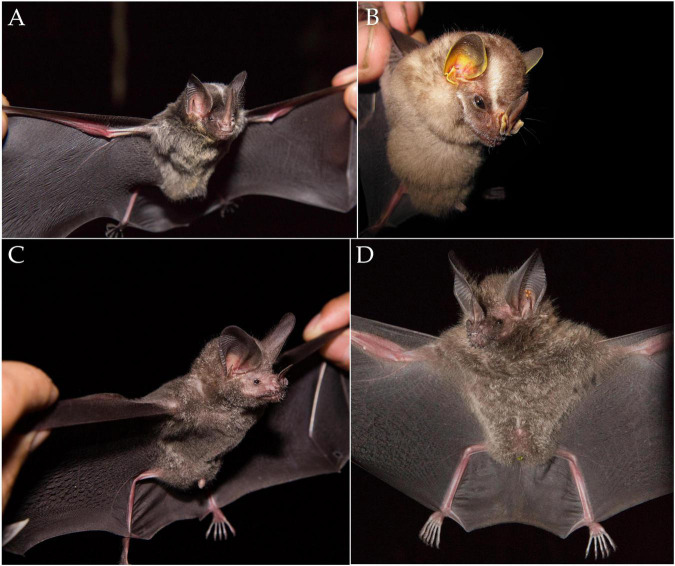
Three new host species for AIV in Phyllostomid bat species: **(A)** *Gardnerycteris crenulatum*, **(B)** *Platyrrhinus brachycephalus*, and **(C,D)** *Trachops cirrhosus*.

The results presented here show evidence, for the first time, of the presence of AIV in the Yangochiroptera bat suborder from Colombia, using molecular approaches. Is worth mentioning that three out of the seven positive neotropical bat species had not been reported as AIV prone hosts before (Figure 3). Thus, this is not only the first AIV record in Colombian, bats but also the first worldwide report of AIV in these three species, expanding the brief list of naturally susceptible bat species to AIV. Taking advantage of the high diversity of bats in the neotropical region where the country is located, the study allowed us to detect AIV in four unreported bat genera to date: *Carollia* spp., *Gardnerycteris* spp., *Platyrrhinus* spp., and *Trachops* spp. Additionally, neotropical bat species previously reported as seropositive to AIV (Tong et al., 2013) were also found positive for AIV presence by the molecular approaches used in this study (*A. lituratus*, *A. planirostris*, *C. brevicauda*, and *C. perspicillata*). Likewise, AIV presence was detected in two Great fruit-eating bats (*Artibeus lituratus*) and a Flat-faced fruit-eating bat (*Artibeus planirostris*) in this study. In agreement with our results, influenza virus presence has been reported previously in *Artibeus* spp. by serological and molecular methods in different sampling areas and genus species (Supplementary Table 1). On the other hand, IgG serological reactivity against recombinant hemagglutinin 17 subtype (rH_17_) has been detected in *A. jamaicensis*, *A. lituratus*, *A. phaeotis*, and *A. obscurus* in Guatemala, and *A. obscurus*, *A. planirostris*, and *A. lituratus* in Peru (Tong et al., 2013). AIV H_18_N_11_ subtype has also been identified by PCR for the first time in Peruvian *Artibeus planirostris*, and *Artibeus obscurus* in Bolivia (Tong et al., 2013; Liang et al., 2015). The absence of information about AIV and/or influenza-like subtypes in Colombian bats provides more value to the findings shown in the present study as there are no reports or surveillance data available on this subject at the time the study was conducted. There is also a lack of indexed information in the Influenza Research Database (IRD) and the Database of Bat-associated virus (DBatVir), where no sequences of any viral family distinct to *Rhabdoviridae*, *Flaviviridae*, and *Togaviridae* have been reported in Colombian bat species (Squires et al., 2012; Chen et al., 2014). Therefore, the results presented here contribute to the knowledge, providing insights on the relevance that different bat species and particularly the *Artibeus* spp, genus could have in the AIV eco-biology and epidemiology of AIV among neotropical chiropterans. It also helps to illuminate the unclear situation of these viral agents in the new world and the distribution of AIV in neotropics.

A detection in bat oropharyngeal swabs, not previously reported, highlights the importance and makes the virological evidence found in the present study another relevant finding, where 60% (6/10) of the positive AIV samples corresponded to oropharyngeal swabs. These results show not only the feasibility of AIV molecular detection in this type of sample but also draw attention to the potential eco-biological consequences that imply, considering the aerosol transmission of influenza viruses (Cáceres et al., 2021) and the relevance and the pivotal role that fruit-eating bats of genus *Artibeus* sp. could have in AIV eco-epidemiology. Even though the behavior of bats and AIV-bat interactions are not fully elucidated, it is valuable to consider that droplets and/or respiratory aerosols are the main transmission routes of AIV (Richard and Fouchier, 2016) and represent a potential risk of infection to naïve hosts, which could favor adaptation events of the cross-species jump. On the other hand, even if non-virological evidence of H_17_N_10_ and H_18_N_11_ subtypes were found, it is highly recommended to further study and establish AIV subtypes circulating in neotropical bats. In addition, the absence of H_17_N_10_/H_18_N_11_ subtypes molecular detection also opens the possibility that hypothetically divergent lineage of influenza-*like* viruses could be circulating in new unreported hosts and reinforces the enzootic distribution of AIV in neotropical bats (Liang et al., 2015). Molecular approaches like next-generation sequencing would be of major help in clarifying this issue, however, as this was not the purpose of the study, it would be the next step to follow.

Finally, in addition to new worldwide species and sample types reported here for AIV detection, this study allowed to generate of a biobank of molecular grade-preserved samples from various species of Yangochiroptera bats, providing a potential source for molecular identification of diverse infectious agents circulating within bat populations. This is relevant considering that outbreaks, emergence, and the e-emergence of infectious diseases tend to originate from wildlife under anthropic pressure that increases animal/human contact (Uribe-Soto et al., 2020). The results of this study encourage further evaluation of the role that neotropical bats could play in the epidemiology and dissemination dynamics of AIV in chiropteran populations, emphasizing the biotic and microbiological relevance of this unique flying mammalian order. The study and evaluation of pathogen reservoirs helps to understand and to establish preventive measures to limit the risk of dissemination and emergence of infectious diseases under one health principle. Therefore, studies to detect the plethora of viral agents in South American bats are needed.

## Data Availability Statement

The original contributions presented in the study are included in the article/Supplementary Material, further inquiries can be directed to the corresponding author.

## Ethics Statement

The animal study was reviewed and approved by the Bioethics Committee of the Faculty of Veterinary Medicine and Zootechnies of Universidad Nacional de Colombia through code number CB 119-16.

## Author Contributions

MU, GR-N, and MR-P: conceptualization and funding acquisition. MU and MR-P: methodology, visualization, and data curation. MU: software, formal analysis, investigation, and writing—original draft preparation. GR-N and MU: validation and writing—review and editing. MR-P and GR-N: resources. GR-N: supervision and project administration. All authors have read and agreed to the published version of the manuscript.

## Conflict of Interest

The authors declare that the research was conducted in the absence of any commercial or financial relationships that could be construed as a potential conflict of interest.

## Publisher’s Note

All claims expressed in this article are solely those of the authors and do not necessarily represent those of their affiliated organizations, or those of the publisher, the editors and the reviewers. Any product that may be evaluated in this article, or claim that may be made by its manufacturer, is not guaranteed or endorsed by the publisher.

## Footnotes

1 http://www.mgc.ac.cn/DBatVir/

2
http://www.rdml.org
