# Supplementary material for: Molecular Evidence of Orthomyxovirus Presence in Colombian Neotropical Bats
Source: Front Microbiol. 2022 Apr 26;13:845546. doi: 10.3389/fmicb.2022.845546 (PMC9087557; doi:10.3389/fmicb.2022.845546)
Supplement: Supplementary file 1 [file Table_1.DOCX]

Supplementary Material

# Table S1: Scientific and common names of Fruit-eating bat species.

| **Species** | **Common name** |
| --- | --- |
| *Artibeus obscurus* | Dark Fruit-eating Bat |
| *Artibeus planirostris* | Flat-faced Fruit-eating Bat |
| *Artibeus amplus* | Large Fruit-eating Bat |
| *Artibeus hirsutus* | Hairy Fruit-eating Bat |
| *Artibeus schwartzi* | - |
| *Artibeus aequatorialis* | - |
| *Artibeus lituratus* | Great Fruit-eating Bat |
| *Artibeus inopinatus* | Honduran Fruit-eating Bat |
| *Artibeus jamaicensis* | Jamaican Fruit-eating Bat |
| *Artibeus fraterculus* | Fraternal Fruit-eating Bat |
| *Artibeus concolor* | Brown Fruit-eating Bat |
| *Artibeus fimbriatus* | Fringed Fruit-eating Bat |
| *Artibeus phaeotis*^a^ | Pygmy Fruit-eating Bat |
| *Artibeus watsoni*^b^ | Thomas's Fruit-eating Bat |
| *Artibeus tolteca*^c^ | Toltec Fruit-eating Bat |
| *Artibeus cinerea*^d^ | Gervais's Fruit-eating Bat |
| *Artibeus anderseni*^e^ | Andersen's Fruit-eating Bat |
| *Artibeus hartii*^f^ | Velvety Fruit-eating Bat |
| *Artibeus gnoma*^g^ | Dwarf Fruit-eating Bat |
| *Artibeus rosenbergi*^h^ | Rosenberg’s Fruit-eating Bat |
| *Artibeus glauca*^i^ | Silver Fruit-eating Bat |
| *Artibeus azteca*^j^ | Aztec Fruit-eating Bat |

^a^: Synonym *Dermanura phaeotis.* ^b^: Synonym *Dermanura watsoni*. ^c^: Synonym *Dermanura tolteca*. ^d^: Synonym Synonym ^e^: *Dermanura anderseni*. ^f^: Synonym *Enchisthenes hartii*. ^g^: Synonym *Dermanura gnoma*. ^h^: Synonym *Dermanura rosenbergi*. ^i^: Synonym *Dermanura glauca*. ^j^: Synonym *Dermanura azteca*.

**
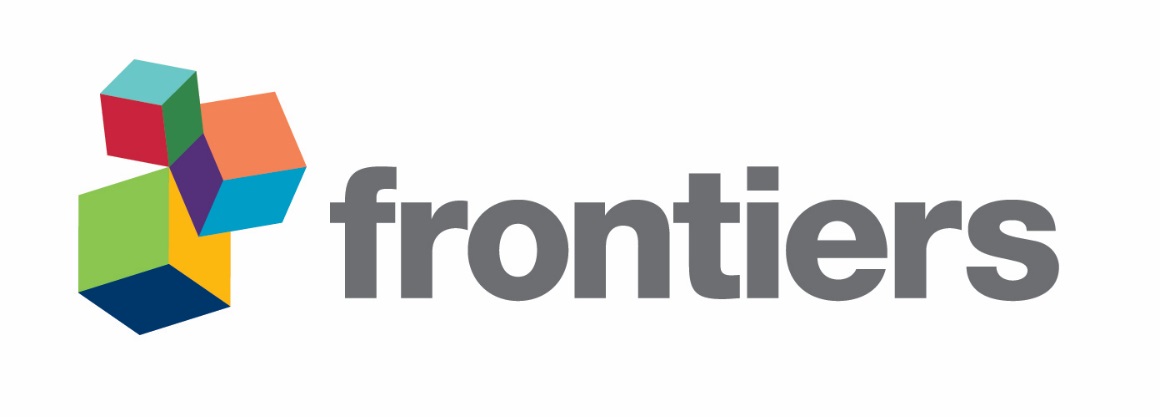
**
